# Supplementary material for: Holistic bursting cells store long-term memory in auditory cortex
Source: Nat Commun. 2023 Dec 7;14:8090. doi: 10.1038/s41467-023-43620-5 (PMC10703882; doi:10.1038/s41467-023-43620-5)
Supplement: Supplementary file 1 — Supplementary Information [file 41467_2023_43620_MOESM1_ESM.pdf]

Holistic Bursting Cells Store Long-term Memory in Auditory Cortex

Supplementary Information

Supplementary information contains 4 supplementary Figures.

Supplementary Figures

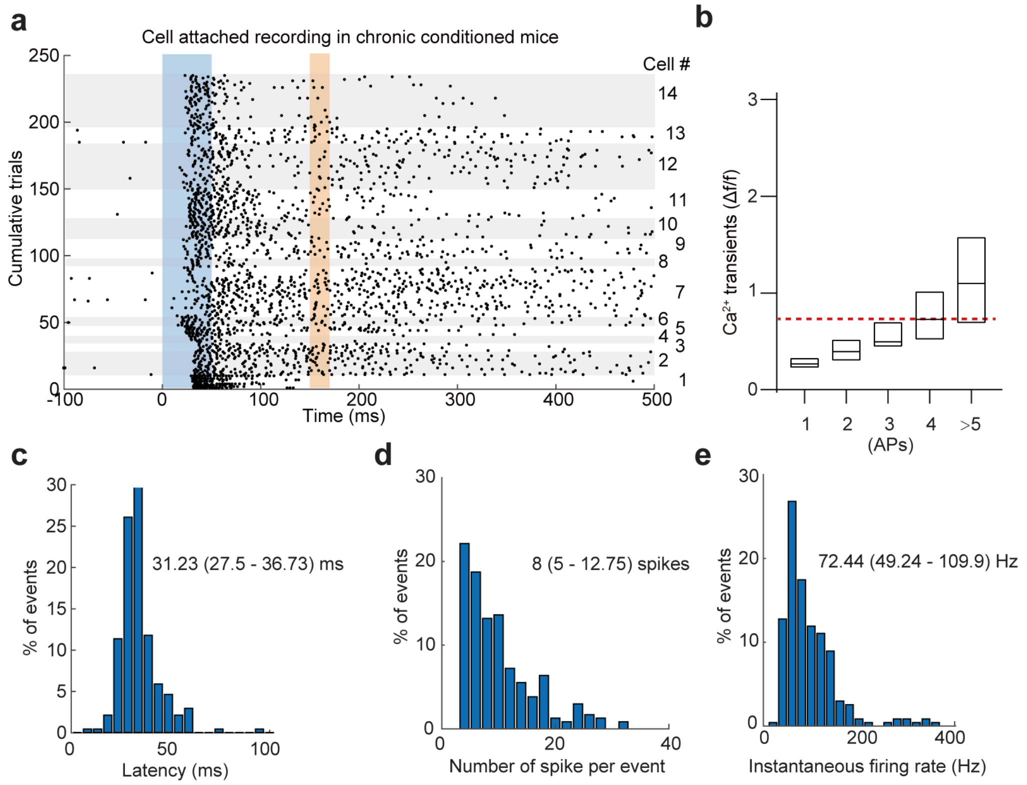

Supplementary Fig. 1 | Single-cell electrophysiology in trained, awake behaving mice. a, Spike raster plot of all trial events for 14 HB cells. **b**, Calibration graph for the GCaMP6m-reported Ca^{2+} response versus number of spikes per sound stimulus response event. Red dashed line indicates the threshold for defining bursting response (lower-bound estimate of response event consisting of ⩾3 spikes). **c**, Histogram of response latency for all the response events as in (a). **d**, Histogram of the number of spikes per burst event for all the response events as in (a). **e**, Histogram of instantaneous firing rate for all the response events as in (a).

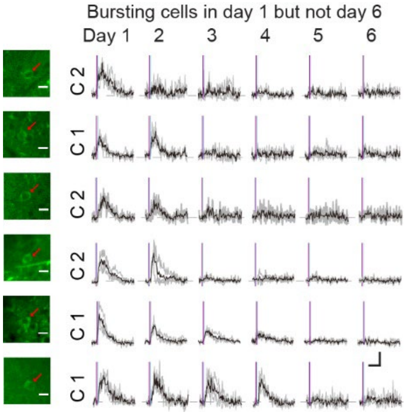

Supplementary Fig. 2 | Example cells that were bursting responsive to a training chord on day 1 but lost bursting responsiveness through the course of training. Single-trial signals (gray) overlaid with trial-averaged signal (black). Purple vertical bar indicates sound stimulus timing. Scale bars: 1.0 Δf/f (vertical) & 1 s (horizontal). C1: Chord 1; C2: Chord 2. Among 1862 cells pooled from 13 animals, on day 1 there were altogether 6 cells exhibiting bursting to chord 1 and 17 cells for chord 2, out of which, 5/6 cells for chord 1 and 8/17 cells for chord 2 had lost their chord-evoked bursting responsiveness during the subsequent training days.

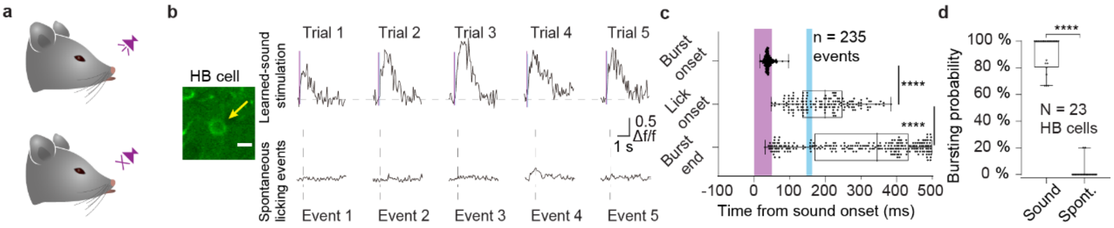

Supplementary Fig. 3 | Activation of HB cells is required for conducting learned sound-evoked licking. **a**, The cartoon of sound-evoked licking (Upper) and spontaneous licking (Lower). Credits of Ms. Jia Lou. **b**, An example HB cell firing pattern at learned-sound stimulation events (upper) and spontaneous licking events (lower).Scale bar: 10 µm **c,** Comparison of the timing of burst onset, burst end, and lick onset. Lick onset vs burst onset, ****, P = 1.76e-12, Mann-Whitney U: 94, two-sided Mann Whitney test; Lick onset vs burst end, ****, P = 6.74e-61, Mann-Whitney U: 10115; 235 burst events, 150 lick events, two-sided Mann Whitney test. **d,** Bursting probability of HB in sound-evoked licking and spontaneous licking events. ****, p = 1.97e-018, t_{22}=27.21, two-sided paired t-test.

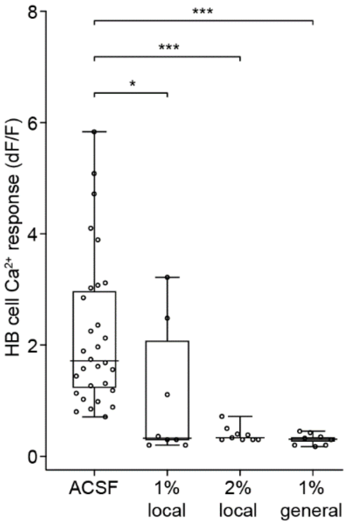

Supplementary Fig. 4 | Test and analysis of Ca^{2+} response of HB cells for different methods of intervention. ACSF: artificial cerebral-spinal fluid; 1% or 2% local: 1% or 2% v/v local aqueous application of isoflurane, (see methods); 1% general: gaseous general inhalation of 1% v/v isoflurane (anesthesia). ACSF vs. 1% local, *, P = 0.0306; ACSF vs. 2% local, ***, P =0.0003; ACSF vs. 1% general, ***, P = 0.0003. $F_{(3, 51)}$ = 10.39, One-way anova test and Tukey post-test.
