# Supplementary material for: Holistic bursting cells store long-term memory in auditory cortex
Source: Nat Commun. 2023 Dec 7;14:8090. doi: 10.1038/s41467-023-43620-5 (PMC10703882; doi:10.1038/s41467-023-43620-5)
Supplement: Supplementary file 3 — Reporting Summary [file 41467_2023_43620_MOESM3_ESM.pdf]

Corresponding author(s): NCOMMS-23-10060A

Last updated by author(s): Mar 9,2023

Reporting Summary

Nature Portfolio wishes to improve the reproducibility of the work that we publish. This form provides structure for consistency and transparency in reporting. For further information on Nature Portfolio policies, see our Editorial Policies and the Editorial Policy Checklist.

Statistics

For all statistical analyses, confirm that the following items are present in the figure legend, table legend, main text, or Methods section.

n/a	Confirmed	
☐	☒	The exact sample size (*n*) for each experimental group/condition, given as a discrete number and unit of measurement
☐	☒	A statement on whether measurements were taken from distinct samples or whether the same sample was measured repeatedly
☐	☒	The statistical test(s) used AND whether they are one- or two-sided<br>*Only common tests should be described solely by name; describe more complex techniques in the Methods section.*
☒	☐	A description of all covariates tested
☐	☒	A description of any assumptions or corrections, such as tests of normality and adjustment for multiple comparisons
☐	☒	A full description of the statistical parameters including central tendency (e.g. means) or other basic estimates (e.g. regression coefficient) AND variation (e.g. standard deviation) or associated estimates of uncertainty (e.g. confidence intervals)
☐	☒	For null hypothesis testing, the test statistic (e.g. *F*, *t*, *r*) with confidence intervals, effect sizes, degrees of freedom and *P* value noted<br>*Give P values as exact values whenever suitable.*
☒	☐	For Bayesian analysis, information on the choice of priors and Markov chain Monte Carlo settings
☒	☐	For hierarchical and complex designs, identification of the appropriate level for tests and full reporting of outcomes
☒	☐	Estimates of effect sizes (e.g. Cohen's *d*, Pearson's *r*), indicating how they were calculated

Our web collection on statistics for biologists contains articles on many of the points above.

Software and code

Policy information about availability of computer code

Data collection	Two-photon imaging data were collected by custom software written in LabVIEW 2017 (National Instruments) and Electrophysiological data were collected by PatchMaster v2×65 (HEKA Elektronik).The animal's licking behavior was monitored by an infrared camera (frame rate 30 Hz).
Data analysis	We analyzed our two-photon imaging data using LabVIEW 2017 (National Instruments), then analyzed by Igor Pro 6.0 (Wavemetrics) and custom software written in Matlab 2018b (Mathworks). Image J 1.51(NIH) was used for image processing. Prism 8.4 (GraphPad) was used for data statistical analyses. A custom-written software written in MATLAB 2018 was used to extract the tongue movements and determine the timepoint when the tongue touches a water spout (left or right).

For manuscripts utilizing custom algorithms or software that are central to the research but not yet described in published literature, software must be made available to editors and reviewers. We strongly encourage code deposition in a community repository (e.g. GitHub). See the Nature Portfolio guidelines for submitting code & software for further information.

Data

Policy information about availability of data

All manuscripts must include a data availability statement. This statement should provide the following information, where applicable:

- Accession codes, unique identifiers, or web links for publicly available datasets
- A description of any restrictions on data availability
- For clinical datasets or third party data, please ensure that the statement adheres to our policy

Large chronic raw image data from wide-field imaging and two-photon imaging are available upon request from the authors. No data sets that require mandatory deposition into a public database were generated during the current study. Source data underlying Figs. 1–5 and Supplementary Figs. 1-5 are available as a Source data file.

Human research participants

Policy information about studies involving human research participants and Sex and Gender in Research.

Reporting on sex and gender	Human research is not involved in this study
Population characteristics	None.
Recruitment	None.
Ethics oversight	None.

Note that full information on the approval of the study protocol must also be provided in the manuscript.

Field-specific reporting

Please select the one below that is the best fit for your research. If you are not sure, read the appropriate sections before making your selection.

☒ Life sciences ☐ Behavioural & social sciences ☐ Ecological, evolutionary & environmental sciences

For a reference copy of the document with all sections, see nature.com/documents/nr-reporting-summary-flat.pdf

Life sciences study design

All studies must disclose on these points even when the disclosure is negative.

Sample size	No statistical methods were used to predetermine sample sizes. Samples sizes were determined based on our own previous studies (Chen et al, Nature, 2011; Wang et al, Nature Communications, 2020) about using behavioral, two-photon imaging and electrophysiological recording approaches. Samples sizes adopted in this study were sufficient for detecting robust effects.
Data exclusions	In the process of chronic experiment, these data excluded from that interrupt the experiment due to infection, anxiety and other reasons should be excluded
Replication	All behavioral, chronic two-photon imaging and electrophysiological experiments were performed over at least 6 independent replicates. The study effects were all successfully replicated in the experiments.
Randomization	For all experiments, samples were randomized where appropriate for data collection and analysis.
Blinding	In the studies, investigators were blinded to groups for data collection and analysis.

Reporting for specific materials, systems and methods

We require information from authors about some types of materials, experimental systems and methods used in many studies. Here, indicate whether each material, system or method listed is relevant to your study. If you are not sure if a list item applies to your research, read the appropriate section before selecting a response.

Materials & experimental systems

n/a	Involved in the study
☒	☐ Antibodies
☒	☐ Eukaryotic cell lines
☒	☐ Palaeontology and archaeology
☐	☒ Animals and other organisms
☒	☐ Clinical data
☒	☐ Dual use research of concern

Methods

n/a	Involved in the study
☒	☐ ChIP-seq
☒	☐ Flow cytometry
☒	☐ MRI-based neuroimaging

Animals and other research organisms

Policy information about studies involving animals; ARRIVE guidelines recommended for reporting animal research, and Sex and Gender in Research

Laboratory animals	C57BL/6J male mice (2-3 months old) were provided by the Laboratory Animal Center of the Third Military Medical University. The mice were housed in a temperature (22-25 °C) and humidity-controlled (50-60% relative humidity) room on a cycle of 12 h light/dark (lights off at 19:00).
Wild animals	No wild animals were used in this study.
Reporting on sex	Male.
Field-collected samples	No field-collected samples were used in this study.
Ethics oversight	Third Military Medical University Animal Care and Use Committee.

Note that full information on the approval of the study protocol must also be provided in the manuscript.
